# COVID‐19: Moving beyond the pandemic

**DOI:** 10.1111/jan.14438

**Published:** 2020-07-14

**Authors:** Joanne McPeake, Natalie Pattison

**Affiliations:** ^1^ University of Glasgow Glasgow UK; ^2^ University of Hertfordshire School of Health and Social Work London UK

## 1 INTRODUCTION

Severe acute respiratory syndrome Coronavirus 2 (SARS‐CoV‐2) is the cause of COVID‐19. As of June 1st 2020, there were over 6 million cases of COVID‐19 internationally and over 370,000 deaths (John Hopkins University, 2020). There has been significant effort to increase hospital and healthcare capacity to reduce the number of fatalities associated with this global pandemic (Choi & Logsdon, 2020). Public health measures have been universally enforced, including the use of social distancing and self‐isolation for those most at risk. Due to this appropriate high demand in the acute phase of this pandemic, the long‐term sequalae of COVID‐19 has had less attention, and clinically much less focus.

## 2 WHAT ARE THE POTENTIAL LONG‐TERM CONSEQUENCES OF COVID‐19?

Learning from different disciplines suggests the long‐term sequalae of severe infection, and the problems associated with the public health measures used to slow the rate of infection of COVID‐19, will be multifaceted and will likely encompasses emotional, physical and social problems.

Governments across the globe are actively placing countries in ‘lockdown’ and asking citizens to socially isolate to prevent the spread of the COVID‐19. This, alongside the social isolation faced by hospitalized patients who are not permitted visitors, could lead to, or exacerbate mental health and emotional problems (Usher, Bhullar, & Jackson, 2020). Social isolation is an independent risk factor for mortality and should not be underestimated as a contributor to excess mortality. This was highlighted in a large meta‐analysis of almost 150 studies, which revealed social isolation had a risk profile similar to that of well‐established physical risk factors, such as obesity and smoking (Holt‐Lunstad, Smith & Layton, 2010).

The physical consequences for those who are infected could be significant. This is especially true of patients who require treatment in the Intensive Care Unit (ICU), which is estimated to be approximately 10% of all patients who are actively infected (Remuzzi & Remuzzi, 2020). Long‐term physical problems following critical care are common and include poor balance, muscle weakness, chronic pain and fatigue (Devine et al., 2019). These problems may be particularly exacerbated in patients with COVID‐19, as the treatment pathway often includes increased sedation usage and muscle relaxants, resulting in limited active mobilization: two activities (or lack thereof) which have direct implications for long‐term physical and cognitive outcomes in critically ill patients (Pun et al., 2019).

The long‐term social consequences of COVID‐19 could also be catastrophic and have major implications for health. This pandemic is likely to bring financial recessions in many countries, with mass unemployment already reported in areas such as the USA (OECD, 2020). Decades of evidence have shown the socioeconomic gradient which poor health takes; as such we may see widening health inequalities during this pandemic. The implementation of social distancing is also a mechanism by which socioeconomic health inequalities could increase across all generations. Many people on low pay or those who are self‐employed may struggle to cope without income for many months; this may result in people risking their health to ensure that they have an adequate income. School closures are likely to have a disproportionate negative impact on attainment and well‐being for children living in socioeconomically deprived areas. Finally, there also appears to be an uneven demographic spread of the virus. For example, ICU admission data from the UK demonstrates that there is a disproportionate number of patients from Black, Asian, Minority and Ethnic groups admitted as a result of COVID‐19 (Intensive Care National Audit & Research Centre, 2020). This combination of social problems is likely to cause long‐lasting health consequences, which could lead to problems for a generation if they are not adequately and sensitively managed.

## 3 WHAT ABOUT FAMILIES?

The short‐term impact of having a family member admitted to hospital with COVID‐19 or supporting a family member who is isolated cannot be underestimated. These individuals are likely to face significant stress and anxiety. This anxiety is exacerbated with the restriction of hospital visitation which has been implemented internationally; families can no longer be present to understand the illness and its trajectory, and even at the end of life, family members may not have the opportunity to be present. We know that these families will experience high rates of post‐traumatic stress disorders and complicated grief (Kentish‐Barnes et al., 2015).

The long‐term consequences for family members of survivors will be challenging. In the critical care literature, it is well documented that family members have to make significant adjustments to working life, with up to 20% having to stop working altogether, due to high care‐giving responsibilities following ICU admission (Griffiths et al., 2013). The role families play in supplementing formal health and social care provision has a significant impact on the aforementioned psychological burdens but also on fatigue, financial burden and impact on working and family life (McPeake et al., 2016).

## 4 IMPROVING OUTCOMES

We have proposed a framework describing potential strategies for supporting patients and families throughout the recovery arc (Table 1). Although not exhaustive, it provides central pillars of care for patients recovering from long‐term hospitalization and critical care following COVID‐19.

**Table 1:** Provision of care for COVID‐19 hospitalised survivors across the recovery arc

	Hospital 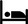	Hospital Discharge 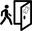	Immediate Community Care 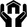	Long‐Term Care 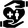
Patient	‐Fundamental patient care	‐Chronic disease support ‐Treatment escalation plans discussed and documented ‐Information provision and expectation management	‐Management of social and welfare issues ‐Appropriate physical, emotional and cognitive support	‐Vocational Rehabilitation ‐Reconnection with care provider ‐Ongoing access to service for physical, emotional and cognitive issues ‐ Readjustment and coping support to reach ‘new normal’
		Provision of timely and adequate communication between community and acute care providers		
Family/Caregivers	‐Provision of timely and accurate communication ‐ Emotional support	‐Provision of information ‐Signposting to community support	‐Assessment and management of mental and emotional health needs ‐Ensuring access to adequate social and welfare support as needed	
Palliative/Bereavement Support and Management				

The implications for nursing care are several‐fold. Firstly, nursing staff are leaders in delivering fundamental care, which can have long‐lasting implications. For example, ensuring patients do not develop pressure areas due to limited mobility and indeed encouraging mobility when safe will improve long‐term physical outcomes and reducing healthcare utilization. Nurses are also key in the management of chronic conditions, which may be exacerbated due to acute illness related to COVID‐19. Patients who move through several transitions of care, which is common with the COVID‐19 patient group, are more likely to have problems with re‐establishment of chronic condition management (MacTavish et al., 2019). Nurses can work across the MDT, coordinating care to ensure the patient care delivered is safe and appropriate in the context of acute illness.

This leadership role in improving long‐term outcome of patients and families also extends into the community setting. Nurses in the community are at the forefront of care delivery; as such they must have an understanding of the long‐term physical, social and emotional problems which this patient group and their family members may have. Although nurses cannot manage, treat or ‘fix’ all of the issues discussed in this piece, they must understand how to sign‐post patients to meaningful resources, many of which will be pre‐existing and based in the third sector, including charities and voluntary organizations. This type of integrated health and social care management has seen success in the critical care recovery setting in the UK and could potentially improve health‐related quality of life in this vulnerable group (McPeake, Devine, MacTavish, Quasim, & T., 2017). Finally, the role of the Public Health Nurse will be crucial in the coming months and years. This group of nurses will be key to ensuring that younger, vulnerable children are adequately supported to reach their full potential in life.

For patients there is the possibility of post‐traumatic growth, a phenomenon seen following serious illness, including post‐critical illness, where reflection on the traumatic and acute illness allows personal growth in relationships, living life to the full, recognizing personal strengths and gaining personal insight (Barskova & Oesterreich, 2009; Connerty & Knott, 2013; O’Gara, Tuddenham, & Pattison, 2018). It aligns to the concept of meaning making following significant life events and allows families and patients to regain control of their lives, which will be crucial to recovery post‐COVID‐19. Nurses are ideally placed to nurture and support this aspect of recovery.

The large numbers of healthcare workers infected, and the toll of caring for high volumes of patients who die from COVID‐19, will also have an impact on both the short‐ and long‐term health and well‐being on healthcare workers. As nurses, we have a critical role in leading the changes needed to support the well‐being of the healthcare workforce, by ensuring we mitigate against burnout and moral injury, and focus on building resilience and at an organizational level (Dewey, Hingle, Goelz & Linzer 2020, Wu, Connors & Everly 2020).

## 5 CONCLUSION

More than ever, we need a competent and confident nursing workforce, who support patients in both the short and long term in these unprecedented times. Nurses also need to have the vision of the likely future health and well‐being implications, driving forward services and healthcare response, to help rebuild the lives of individuals affected.

## CONFLICT OF INTEREST

The authors have no other conflicts of interest.

## FUNDING INFORMATION

Dr Joanne McPeake is funded by a Fellowship from the University of Cambridge (PD‐2019‐02‐16).
